# Complete Genome Sequence of Enterobacter roggenkampii RX.G5M56, a C1-Metabolizing Strain from a Freshwater Stream in Hong Kong

**DOI:** 10.1128/mra.00015-23

**Published:** 2023-03-15

**Authors:** D. Y. Xu, K. M. Leung, G. K. K. Lai, F. C. C. Leung, S. D. J. Griffin

**Affiliations:** a Shuyuan Molecular Biology Laboratory, The Independent Schools Foundation Academy, Hong Kong SAR, China; Indiana University, Bloomington

## Abstract

The C1-metabolizing strain Enterobacter roggenkampii RX.G5M56 was isolated from a freshwater stream in Hong Kong. Its complete genome, a single chromosome of 4,772,201 bp (GC content of 56.05%), was established through hybrid assembly.

## ANNOUNCEMENT

Enterobacter roggenkampii, previously “Hoffmann cluster IV,” is a member of the Enterobacter cloacae complex (ECC) (1). While Enterobacter spp. are recognized as human pathogens (2), E. roggenkampii represents only around 4% of clinical ECC isolates (3–6). Commonly associated with plants (7, 8), it has been proposed for crop growth promotion (9), although its versatile biochemistry includes broad intrinsic antimicrobial resistance (10) and the ability to hasten the corrosion of steel (11). As endophytes and in the rhizosphere, isolates of E. roggenkampii may produce indole-3-acetic acid and siderophores, solubilize phosphate, and fix nitrogen (7, 12). Here, isolate RX.G5M56 was found to utilize methanol, a trait known to be enriched within plant-bearing soils (13).

A 50-μL water sample collected from a freshwater stream in Hong Kong (22.256167N, 114.136222E) was added to 1 mL of a minimal salts medium (14) containing 5% (vol/vol) methanol (MSM-MeOH) as the only carbon source. Following incubation at 27°C for 7 days with shaking, the mixture was streaked on Luria agar (15) to give cream-colored colonies after overnight growth. Selected colonies were passaged to purity on Luria agar, and isolates were subsequently reassessed for growth in MSM-MeOH by plate counts of aliquots withdrawn daily over 5 days. A single colony of RX.G5M56, the isolate showing the strongest growth in MSM-MeOH, was spread on Luria agar and incubated for 24 h before harvesting for DNA extraction (Qiagen DNeasy PowerSoil Pro kit).

Paired-end short-read sequencing libraries were prepared using the NovaSeqX series 10B reagent kit and sequenced via the NovaSeq 6000 platform (SP flow cell, 250-bp paired-end reads, v1.5 reagent kit). Reads were quality filtered and trimmed using TrimGalore! v0.6.7 (https://github.com/FelixKrueger/TrimGalore) (stringency:3, –e:0.2), which produced 1,799,526 read pairs (mean length, 250 bp) totaling ~450 Mbp. Long-read libraries, which were prepared from the same extracted DNA using the rapid barcoding kit SQK-RBK004 without size selection, were sequenced via an Oxford Nanopore Technologies Spot-ON flow cell (vR9.4.1), MinION sequencer, and MinKNOW v20.06.2 software, with base calling by Guppy v6.1.5. The final long-read data set, trimmed with Filtlong v0.2.1 (https://github.com/rrwick/Filtlong) (min_length:2000, target_bases:500Mbp, length_weight:10), totaled 81,315 reads (502 Mbp), with a mean length of 6,149 bp (*N*_50_, 7,640 bp). Default parameters were used for all software unless otherwise specified.

The complete genome sequence combined Illumina and MinION datasets using Unicycler v0.4.3, which yielded a circular chromosome of 4,772,201 bp (GC content, 56.05%; mean coverage, 105×); the sequence was submitted to NCBI PGAP v5.0 and to PATRIC-BRC for annotation. Mash/MinHash analysis using PATRIC found RX.G5M56 to be closest to Enterobacter roggenkampii strain BP10374 (GenBank accession number CP038471), with an average nucleotide identity (ANI) of 99.91% (ANIb by JSpeciesWS [16]).

A glutathione-dependent formaldehyde detoxification pathway, which is common in *Enterobacteriaceae* strains (17, 18), is likely assisted in RX.G5M56 by a putative *S*-hydroxymethyl-glutathione synthase (EC 4.4.1.22) (18). The AmpC variant MIR-10 (19) is chromosomally encoded, and growth within an initial zone of inhibition after a 24-hour incubation with ertapenem, imipenem, and cefepime test strips (Liofilchem) was consistent with induction of the β-lactamase (20).

### Data availability.

The complete genome sequence and raw sequence data for Enterobacter roggenkampii RX.G5M56 are available through NCBI under BioProject accession number PRJNA875571, with GenBank accession number CP104001 and SRA accession numbers SRX17874418 (MinION) and SRX17874417 (NovaSeq).
